# Supplementary material for: Unzipping of black phosphorus to form zigzag-phosphorene nanobelts
Source: Nat Commun. 2020 Aug 6;11:3917. doi: 10.1038/s41467-020-17622-6 (PMC7411046; doi:10.1038/s41467-020-17622-6)
Supplement: Supplementary file 1 — Supplementary Information [file 41467_2020_17622_MOESM1_ESM.pdf]

Supplementary Information

Unzipping of black phosphorus to form zigzag-phosphorene nanobelts

Liu et al.

Supplementary Information

Unzipping of black phosphorus to form zigzag-phosphorene nanobelts

Zhifang Liu[1,6], Yilin Sun[2,6], Huaqiang Cao[1]✉, Dan Xie[2]✉, Wei Li[3], Jiaou Wang[4] & Anthony K. Cheetham[5]✉

[1]*Department of Chemistry, Tsinghua University, Beijing 100084, China.*

[2]*Institute of Microelectronics, Beijing National Research Center for Information Science and Technology (BNRist), Tsinghua University, Beijing 100084.*

[3]*Center of Rare Earth and Inorganic Functional Materials, National Institute for Advanced Materials, Nankai University, Tianjin 300350, China.*

[4]*Beijing Synchrotron Radiation Facility, Institute of High Energy Physics, Chinese Academy of Sciences, Beijing 100049, China.*

[5]*Department of Materials Science and Metallurgy, University of Cambridge, Cambridge CB3 0FS, U.K.*

[6]*These authors contributed equally: Z. L. and Y. S.*

✉*Corresponding authors (e-mail: hqcao@mail.tsinghua.edu.cn, xiedan@tsinghua.edu.cn & akc30@cam.ac.uk)*

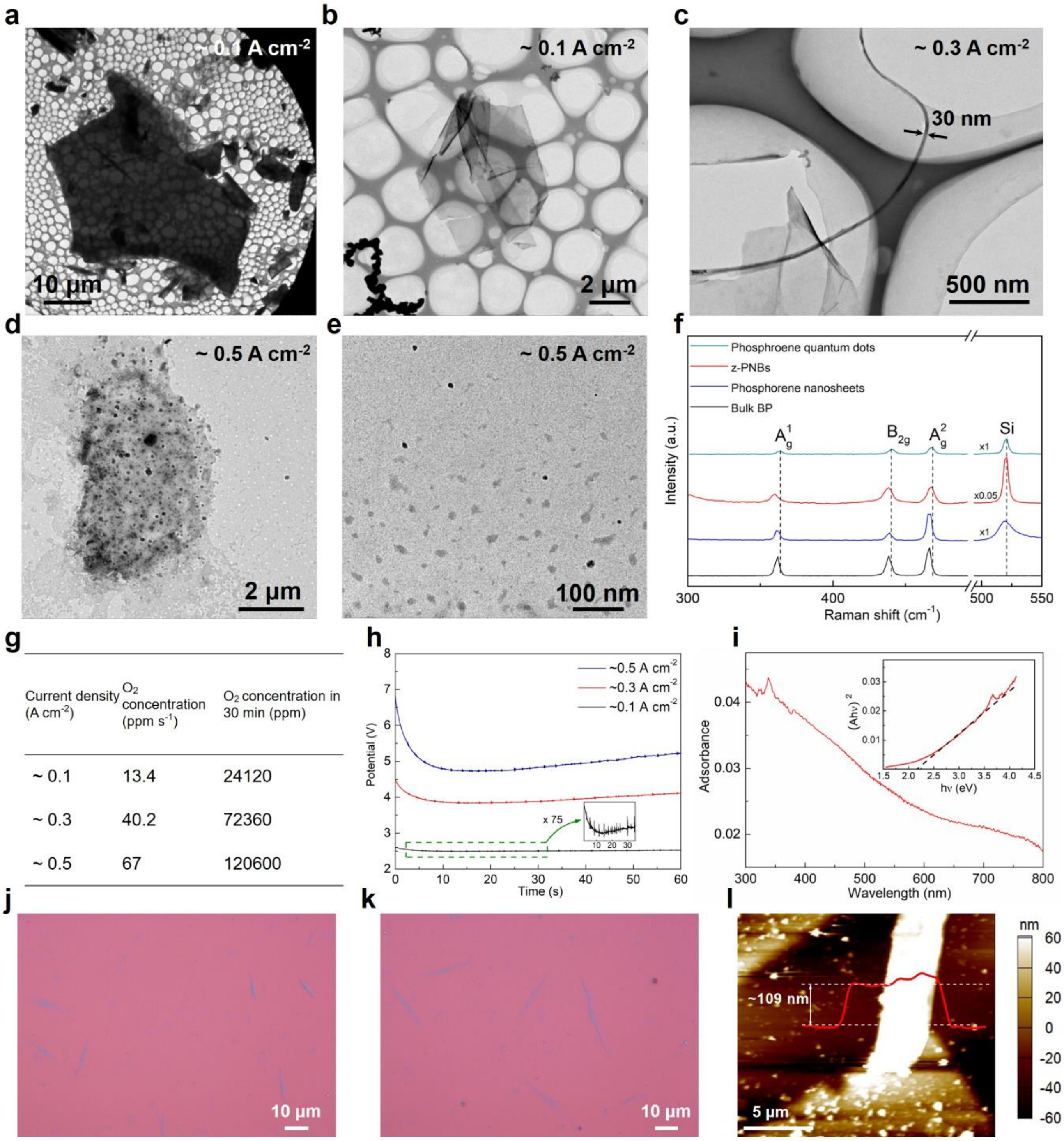

Supplementary Figure 1. Controllable morphology and thickness of phosphorene. **a-b** TEM images of phosphorene nanosheets. **c** TEM images of a narrow phosphorene nanobelt with a width of 30 nm. **d-e** TEM images of phosphorene quantum dots. **f** An example Raman spectrum of phosphorene quantum dots compared to bulk black phosphorus (BP), phosphorene nanosheets and z-PNBs. The results showed three Raman peaks of phosphorene quantum dots at 364.0, 440.3, 468.5 cm^{-1}, respectively. And the intensity ratio of A_g^1 and Si peak is ~0.2, corresponding to about trilayer phosphorene[S1]. **g** Oxygen concentration at different current densities during the exfoliation process. **h** Chronopotentiometry curves during the exfoliation process at different current densities from ~0.1 to 0.5 A cm^{-2}. The resistance increased with longer exfoliation time, leading to the larger bandgap. It is noted that the origin sharp decrease is due to the increased surface area[S2]. Inset: The enlarged 75 times chronopotentiometry curve of ~0.1 A cm^{-2}. **i** Ultraviolet absorption spectrum of z-PNBs solution. Inset: Relative $(Ah\nu)^2$-hν curve of **i**. The optical bandgap could be calculated to be 2.16 eV, higher than that of bulk

BP. **j-k** Comparison of length prepared with different pristine BP chunks. **j** Shorter BP chunk (~1 cm) as pristine materials, leading to z-PNBs with average length of 10-15 μm. **k** Longer BP chunk (> 1.2 cm) as pristine materials, leading to z-PNBs with length up to 40 μm. **l** A typical AFM image of the z-PNB with thickness of ~109 nm prepared through shorter exfoliation time. Note: the scrolls are caused by strain from the bending/twisting of the nanobelts on TEM grid, while the fragments are attributed to ultrasonic treatment after electrochemical exfoliation.

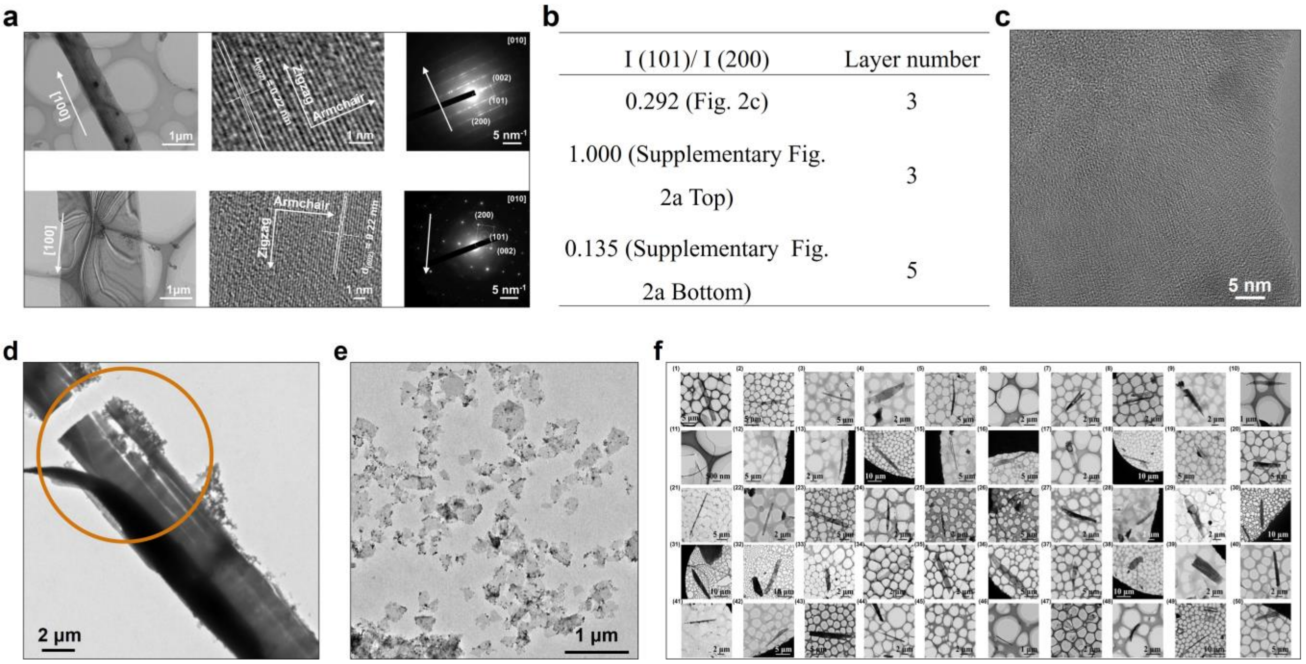

Supplementary Figure 2. TEM characterizations of a series of z-PNBs. a TEM, HRTEM images of z-PNBs and their SAED patterns, showing the nanobelts were unzipped along the zigzag direction. **b** Thickness dependence of the electron diffraction patterns. The intensity ratio between the (101) and (200) reflections is listed. **c** HRTEM image of the edge of z-PNB. It is noted that the edge was absolutely amorphous, indicating that the edge was oxidized. **d** TEM image of a z-PNB without sonication after electrochemical exfoliation. Some branch-like cracks could be observed along one direction. **e** Phosphorene prepared by ultrasonic treatment in the same liquid solution as the reaction system of Fig. 1 in the text or Method without electrochemical exfoliation. **f** TEM images of 73 z-PNBs with different aspect ratios.

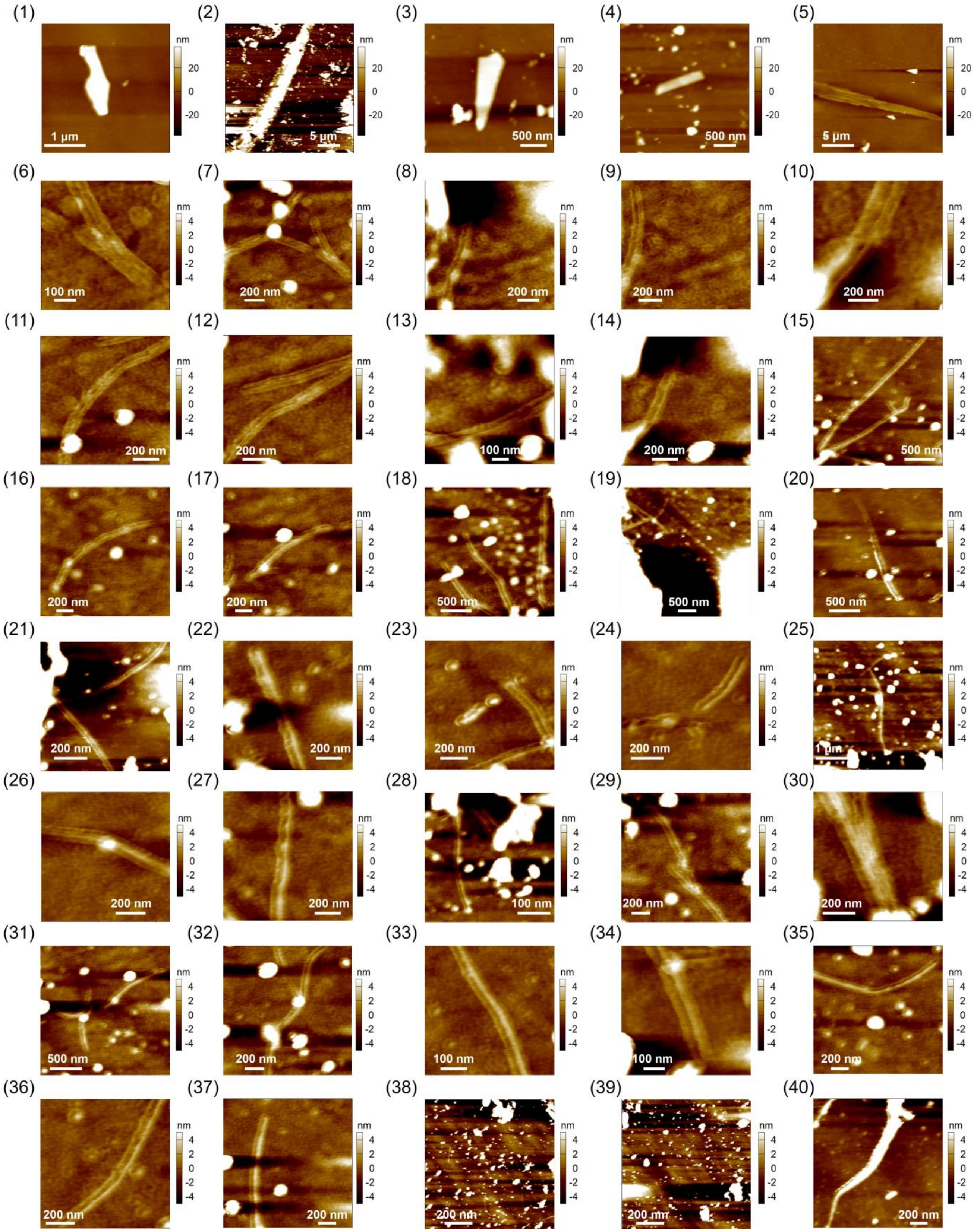

Supplementary Figure 3. AFM image of 56 z-PNBs. The white dots are the fragments generated by ultrasonic treatment after electrochemical exfoliation.

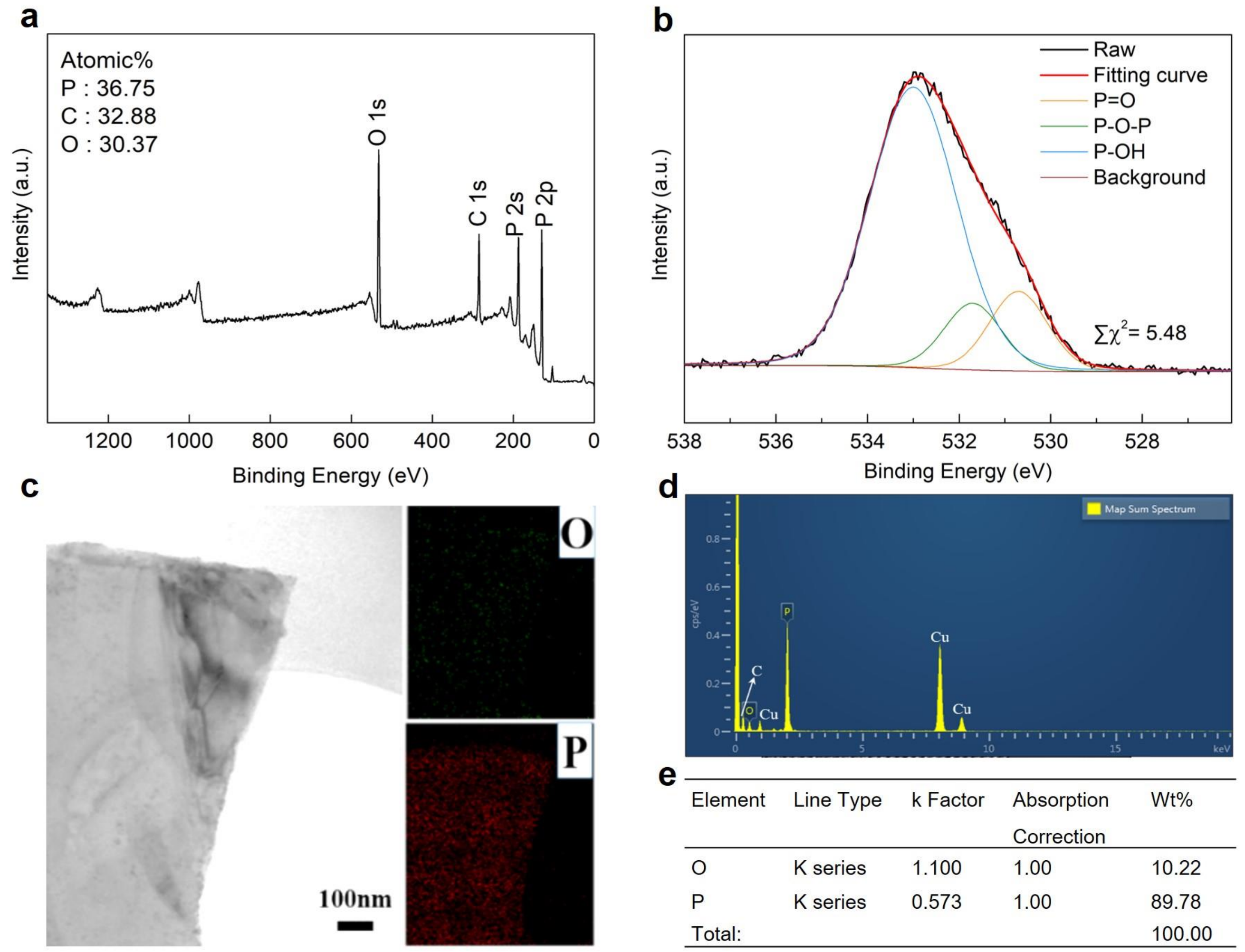

Element	Line Type	k Factor	Absorption Correction	Wt%
O	K series	1.100	1.00	10.22
P	K series	0.573	1.00	89.78
Total:				100.00

Supplementary Figure 4. XPS survey spectrum, O 1s XPS spectra, EDS mapping of z-PNBs and corresponding element content table. a XPS survey spectrum of z-PNBs. **b** High-resolution XPS spectra of the O 1s signal for electrochemically exfoliated z-PNBs, indicating that the content of P-OH (74.26 at.%) P=O (14.04 at.%) and P-O-P (11.70 at.%). **c** EDS mapping of z-PNBs. **d-e** Corresponding element content table. It was noted that additional carbon and copper came from the TEM grid.

Supplementary Table 1. P 2p spilt results and fitting results of peak area.

Type	Binding Energy/eV	Fitting area	Ratio
$2p_{3/2}$	129.7	64356.41	60.07 at.%
$2p_{1/2}$	130.5	32178.21	30.03 at.%
P-OH	133.2	7904.63	7.38 at.%
P_xO_y(including P=O, P-O-P)	134.0	2699.30	2.52 at.%
Fitting peak areas		107138.60	

Background area	105976.60
Error	1.10 %

Supplementary Table 2. O 1s spilt results and fitting results of peak area.

**Type**	**Binding Energy/eV**	**Fitting area**	**Ratio**
P-OH	533.0	60583.82	74.26 at.%
P=O	530.7	11452.89	14.04 at.%
P-O-P	531.7	9543.55	11.70 at.%
Fitting peak areas		81580.26	
Background area		80413.85	
Error		1.45 %	

Supplementary Table 3. XPS peaks with different Lorentzian/Gaussian ratio and relative $\sum\chi^2$. Thus, we select 10% Lorentzian-90% Gaussian to fitting the curves.

Lorentzian (%)	Gaussian (%)	$\sum\chi^2$ (P 2p)	$\sum\chi^2$ (O 1s)
0	100	33.60	24.62
10	90	17.86	5.48
20	80	28.14	7.65
30	70	58.25	14.25
40	60	101.82	23.34
50	50	155.41	37.73
60	40	215.53	52.97
70	30	278.28	69.49
80	20	340.11	93.89
90	10	398.05	112.94
100	0	449.86	131.38

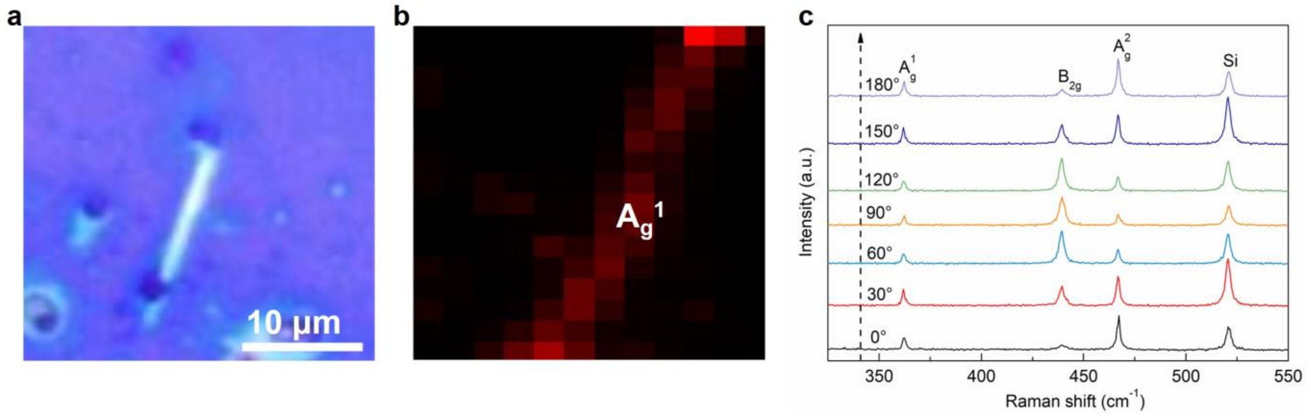

Supplementary Figure 5. Raman mapping and polarize Raman spectra of z-PNB. a Optical image of an individual z-PNB on the 300 nm SiO_2/Si substrate. **b** Raman mapping of **a** with A_g^1 intensity. **c** Polarization-resolved Raman scattering spectra of z-PNB in Fig. 1j with different angles.

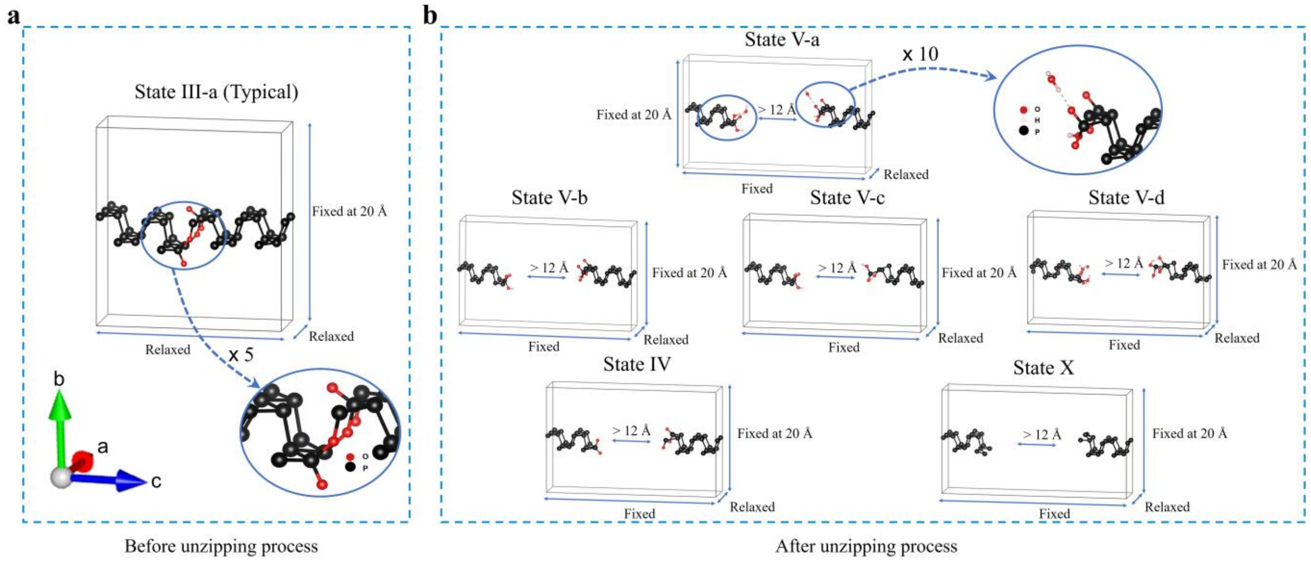

Supplementary Figure 6. Supercell size of typical configurations. a Before unzipping BP structure, a vacuum of ~17.8 Å (*b* = 20 Å) was added in the direction (b axis) normal to the monolayer to avoid spurious interactions between periodic replicas periodic replicas, which could minimize images interactions for the all configurations. **b** After unzipping BP structure, a vacuum of ~17.8 Å (*b* = 20 Å) was added in the direction (b axis) normal to the monolayer, with another direction (c axis) at least 12 Å vacuum after optimization separating the nanobelts to avoid interactions. The black, red, and pink spheres represent phosphorus, oxygen, and hydrogen atoms, respectively. The dotted lines represent hydrogen bond.

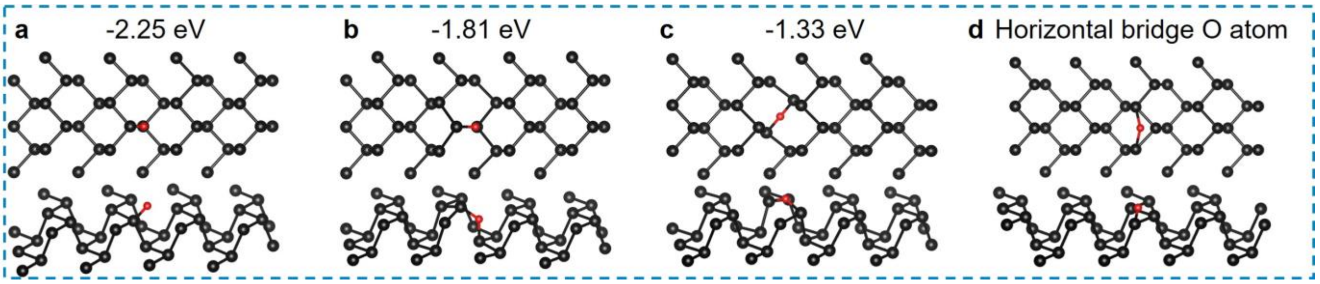

Supplementary Figure 7. Atomic structures and energy of single O atom adsorbed on phosphorene after optimization. a Dangling O atom. **b** Interstitial bridge O atom. **c** Diagonal bridge O atom. **d** Horizontal bridge O atom. The horizontal O bridge atom is calculated to be not a local minima point, O atom will move to the adjacent top site automatically after geometry optimization.

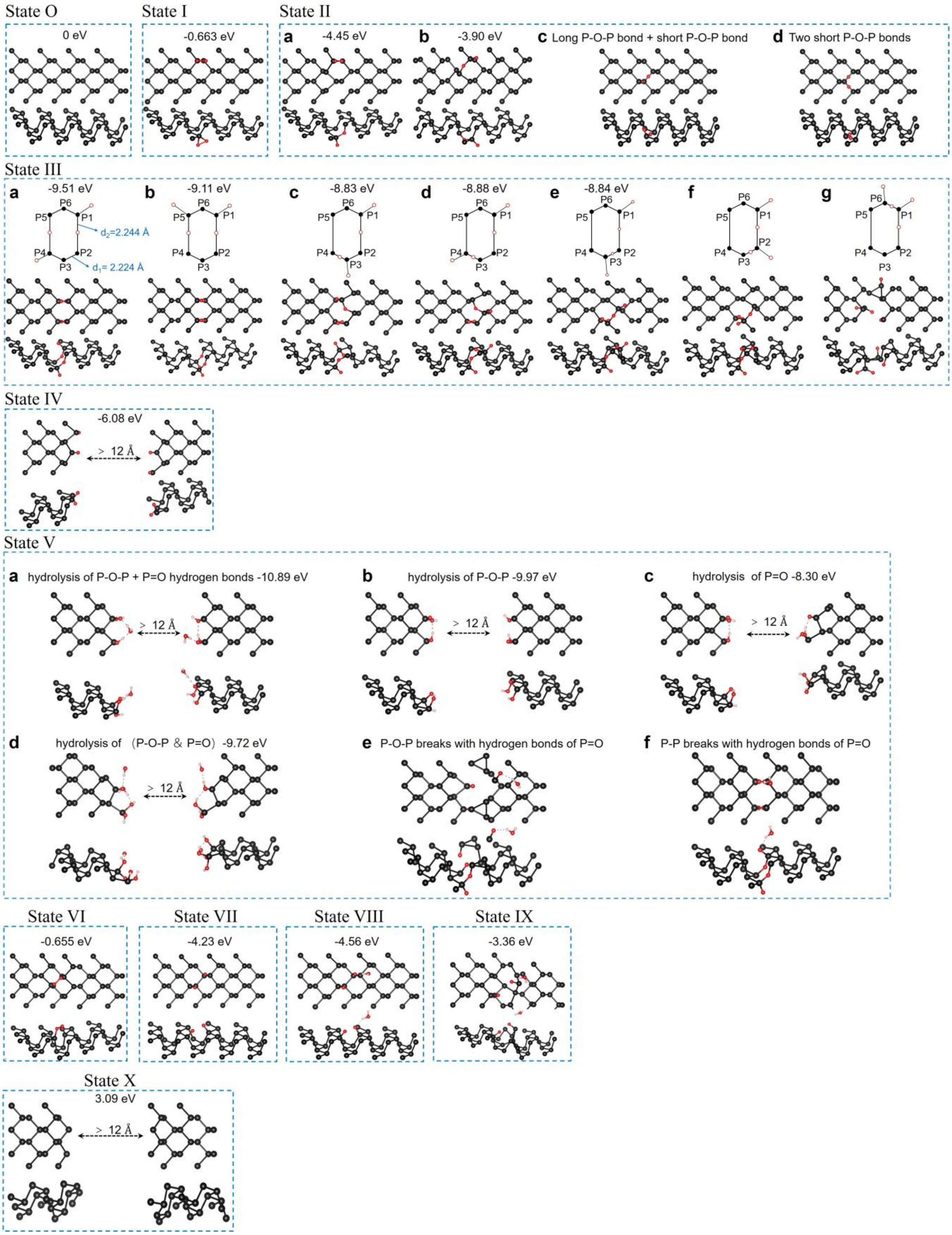

Supplementary Figure 8. Atomic structures and energy of State O-X after optimization. State O: Pristine phosphorene. **Step I:** The first O_2 is chemically adsorbed on a P atom to form an epoxy bond. **Step II:** If the O_2 breaks, forming a P=O dangling bond and a P-O-P

bond: **a** O atom is inserted in the P-P long bond. **b** O atom is inserted in the P-P short bond. Considering that the dissociation of O_2 is just in the form of interstitial O: **c** A long P-O-P bond + a short P-O-P bond. **d** Two short P-O-P bonds. It is noted that the preset configurations of **c** and **d** are not minima points, which will lead to the relaxation of atoms after geometry optimization. **Step III:** first, supposed that the first O_2 combines with the P atom, one O atom is between the P_1 and P_2, and the other O atom forms a P=O dangling bond with P_1. The distance between P_1 and P_2 is d_2, the distance between P_2 and P_3 is d_1, d_1 = 2.224 Å, d_2 = 2.244 Å. The second O_2 is adsorbed on the adjacent side of the first O_2 in seven ways: **a** One O atom is between P_4 and P_5, the other one forms a P=O dangling bond with P_4; **b** One O atom is between P_4 and P_5, the other one forms a P=O dangling bond with P_5; **c** One O atom is between P_3 and P_4, the other one forms a P=O dangling bond with P_3; **d** One O atom is between P_3 and P_4, the other one forms a P=O dangling bond with P_4; **e** One O atom is between P_2 and P_3, the other one forms a P=O dangling bond with P_3; **f,** One O atom is between P_2 and P_3, The other one forms a P=O dangling bond with P_2; **g** One O atom is between P_1 and P_6, The other one forms a P=O dangling bond with P_6. Structures of **f** and **g** have relaxation after optimization, because pristine structure is not the minima point of potential energy surface. **Step IV,** Phosphorene is unzipped without H_2O. H_2O is not considered through this way. **Step V: a** with the hydrolysis of P-O-P bond, hydrogen bond between H_2O and P=O dangling bond, the phosphorene breaks along the zigzag direction. **b** With the hydrolysis of P-O-P bond, the phosphorene breaks along the zigzag direction. **c** With the hydrolysis of P=O dangling bond, phosphorene breaks along zigzag direction. **d** With the hydrolysis of P=O dangling bond and P-O-P bond, phosphorene breaks along zigzag direction. **e** With the hydrogen bond formed between H_2O and P=O dangling bond, P-O-P bond breaks, thus phosphorene breaks along zigzag direction. **f** With the hydrogen bond formed between H_2O and P=O dangling bond, the P-P bond breaks, and the phosphorene breaks along zigzag direction. The structure in **e** and **f** relaxes after optimization, because the present structure is not the minima point of potential energy surface. It is noted that the vacuum between edges of periodic structures in **a, b, c, d** is fixed to more than 12 Å to minimize their interactions after optimization. **Step VI:** Chemical adsorption of O_2 through bridge way. **Step VII:** O_2 dissociation to form two P=O dangling bonds. **Step VIII:** Hydrogen bond is formed between H_2O and P=O dangling bond. **Step IX:** P-P bond breaks with hydrogen bond. **Step X:** Phosphorene breaks without H_2O and O_2. The vacuum between edges of periodic structures are fixed to more than 12 Å to minimize their interactions after optimization.

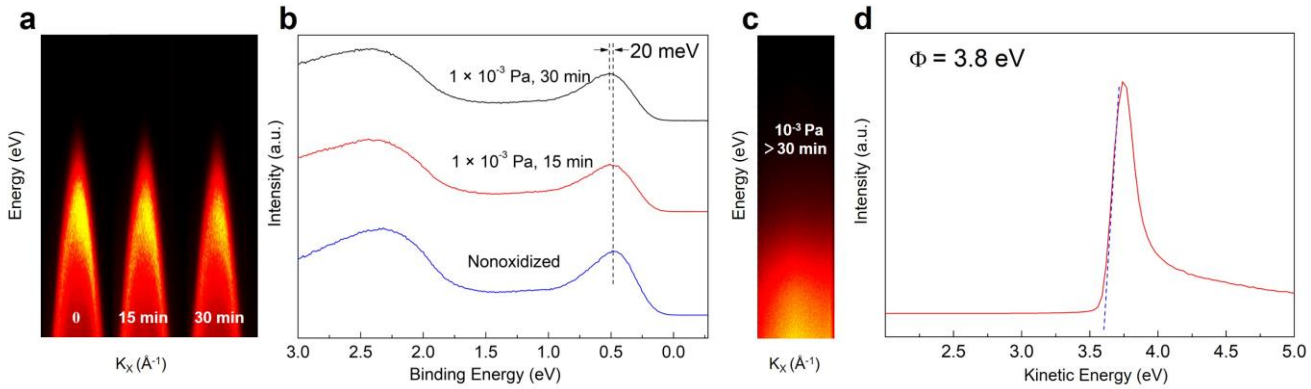

Supplementary Figure 9. Electronic structure of bulk BP and z-PNBs. a Energy versus k dispersion measured by in-situ ARPES for clean BP surface, oxygen-intercalated bulk BP with an oxygen dose of 10^{-3} Pa for 15 and 30 minutes. **b** Energy distribution curves recorded in **a**, showing a tiny variation of 20 meV. **c** Energy versus k dispersion measured by ARPES for clean BP surface, oxygen-intercalated BP with an oxygen dose of 10^{-3} Pa for >30 minutes. **d** UPS spectra evolution at the low kinetic energy region (secondary electron cut off), Φ represents the work function.

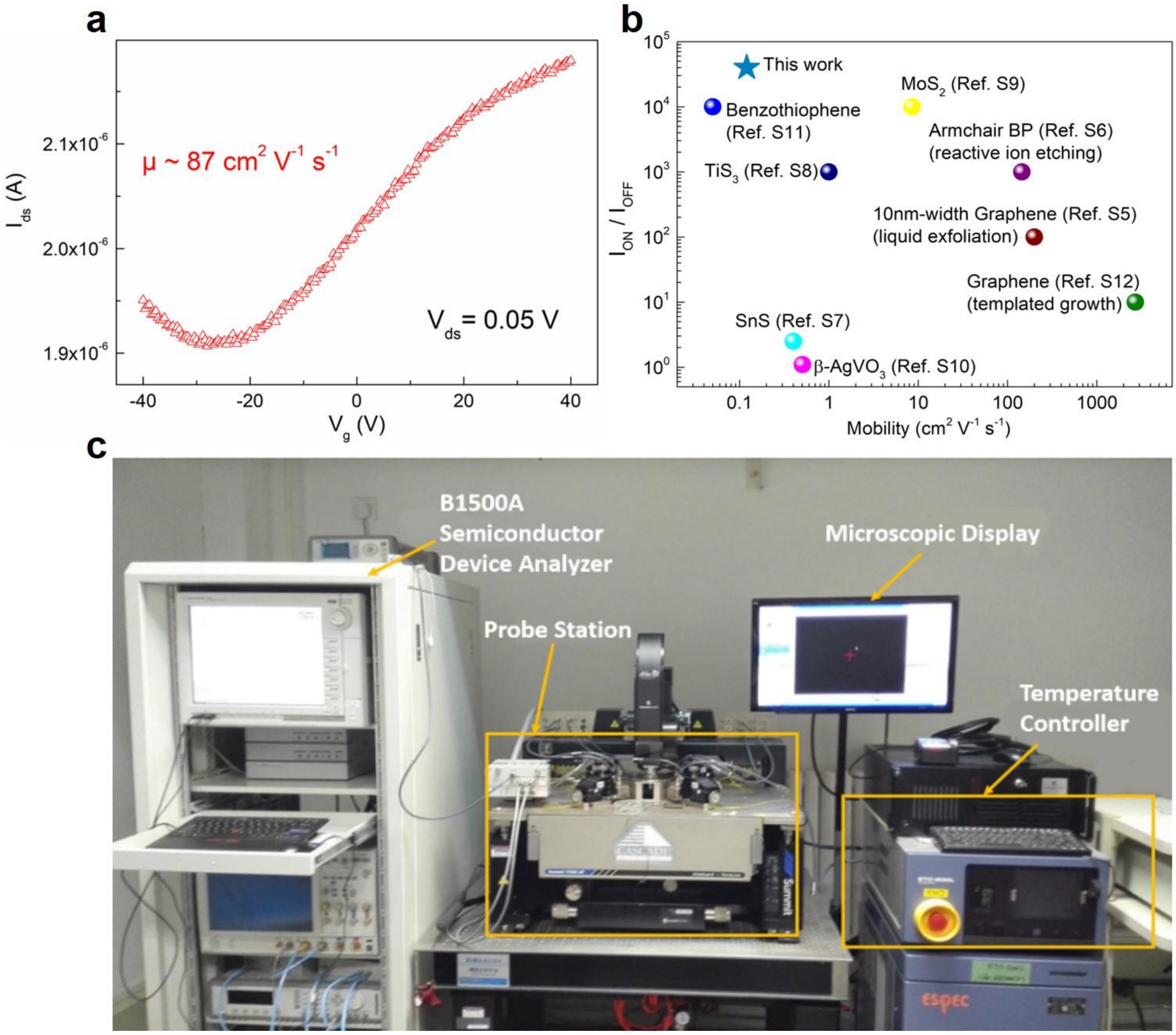

Supplementary Figure 10. Electronic properties of z-PNBs. a Typical I_{ds}-V_g curve of typical n-type electronic behavior with Al (20 nm) and Au (30 nm) as contact metal. The electron mobility (87 $cm^2\ V^{-1}\ s^{-1}$) was among the highest values for n-type BP-based devices [S3,S4], however, the ON/OFF ratio of n-type devices in this work were mostly less than 10 due to the incomplete hole carrier suppression [S3]. **b** Comparison of z-PNB electronic properties we fabricated with other nanoribbon-based devices according to Refs. S5-S12. Besides, the I_{ON}/I_{OFF} of our work was also comparable with that of graphene nanoribbon-based devices ranging from 2 to 10^5, where the hole mobility of these devices has not been discussed[S13-S15]. **c** The Photographs of the electrical measurement B1500A. Each part was also noted in the image by yellow lines.

Supplementary References

S1. Castellanos-Gomez, A. et al. Isolation and characterization of few-layer black phosphorus. *2D Mater.* **1**, 025001 (2014).

S2. Huang, Z. et al. Layer-tunable phosphorene modulated by the cation insertion rate as a sodium-storage anode. *Adv. Mater.* **29**, 1702372 (2017).

S3. Perello, D. J., Chae, S. H., Song, S., & Lee, Y. H. High-performance n-type black phosphorus transistors with type control via thickness and contact-metal engineering. *Nat. Commun*. **6,** 7809 (2015).

S4. Wang, L. et al. Unipolar n-Type conduction in black phosphorus induced by atomic layer deposited MgO. *IEEE Electron Device*

Lett. **40,** 471-474 (2019).

S5. Li, X., Wang, X., Zhang, L., Lee, S. & Dai, H. Chemically derived, ultrasmooth graphene nanoribbon semiconductors, *Science* **319**, 1229-1232 (2008).

S6. Feng, X. et al. High mobility anisotropic black phosphorus nanoribbon field-effect transistor. *Adv. Funct. Mater*. **28**, 1801524 (2018).

S7. Deng, Z. et al. Solution synthesis of ultrathin single-crystalline SnS nanoribbons for photodetectors via phase transition and surface processing. *ACS Nano* **6,** 6197-6207 (2012).

S8. Molina-Mendoza., A. J. et al. High current density electrical breakdown of TiS_3 nanoribbon-based field-effect transistors. *Adv. Funct. Mater*. **27,** 1605647 (2017).

S9. Chen, S. et al. Monolayer MoS_2 nanoribbon transistors fabricated by scanning probe lithography. *Nano Lett.* **19,** 2091-2098 (2019).

S10. Feng, M., Luo, L. B., Nie, B., & Yu, S. H. p-Type beta-silver vanadate nanoribbons for nanoelectronic devices with tunable electrical properties. *Adv. Funct. Mater.* **23,** 5116-5122 (2013).

S11. Pan, Z. et al. Self-assembled π-extended condensed benzothiophene nanoribbons for field-effect transistors. *Chem-Eur. J* **19,** 9771-9774 (2013).

S12. Sprinkle, M. et al. Scalable templated growth of graphene nanoribbons on SiC. *Nat. Nanotechnol.* **5,** 727-731 (2010).

S13. Gao, J. et al. Ambipolar transport in solution-synthesized graphene nanoribbons. *ACS Nano* **10,** 4847-4856 (2016).

S14. Moreno, C. et al. Bottom-up synthesis of multifunctional nanoporous graphene. *Science* **360,** 199-203 (2018).

S15. Llinas, J. P. et al. Short-channel field-effect transistors with 9-atom and 13-atom wide graphene nanoribbons. *Nat. Commun*. **8,** 633 (2017).
